# Sustaining successes and addressing challenges to vaccination – a continued public health mission

**DOI:** 10.2807/1560-7917.ES.2025.30.16.2500284

**Published:** 2025-04-24

**Authors:** Piotr Kramarz, Ines Steffens

**Affiliations:** 1 Scientific Methods and Standards Unit, European Centre for Disease Prevention and Control (ECDC), Stockholm, Sweden; 2 *Eurosurveillance* Editorial Office, European Centre for Disease Prevention and Control (ECDC), Stockholm, Sweden

**Keywords:** immunisation, vaccines, vaccination, public health

The European Immunization Week (EIW) and the World Immunization Week in April are annually recurrent occasions to celebrate the important public health advances reached thanks to the use of vaccines. They are, however, also occasions to remind us of the importance to build on the achievements and the need to sustain them. ‘Vaccine -preventable diseases in humans ─ today's challenges and tomorrow's opportunities’ is the topic of the 2025 annual theme of *Eurosurveillance*. Tying in with the 2025 EIW, this week’s issue of *Eurosurveillance* covers topics such as vaccine-preventable disease outbreaks and outbreak response [1-3] and as the long-term effects of policies aimed at increasing vaccination rates [4]. Three articles specifically focus on measles, highlighting deficiencies in immunity levels [5] and the influence of socioeconomic factors on the spread of the measles virus [2,4]. One article addresses the risk of re-emergence of polio [3].

Data from the European Centre for Disease Prevention and Control (ECDC) monthly report on measles and rubella show that in the 12 months between 1 March 2024 and 28 February 2025, in the European Union/European Economic Area (EU/EEA), more than 35,000 people had measles, a 10-fold increase compared with 2023 when just under 4,000 measles cases were reported, with continued transmission occurring in several European countries in 2025 [6]. Tragically, there were also fatalities caused by measles in Europe, and measles-related deaths have also been reported in conjunction with sustained measles transmission in Texas, United States (US) [7]. The US is one example of a high-income setting where measles had been declared eliminated in 2000 thanks to vaccination efforts [8,9]. In Europe, the ongoing surge of measles followed a period of unusually low measles activity during the COVID-19 pandemic, and a further rise in numbers is anticipated in spring/summer 2025.

Measles stands out as one of the most contagious diseases, with a basic reproductive number of 12–18. It spreads easily through the air, particularly in communities where vaccination rates are low. Among those diagnosed with measles in the EU/EEA between the beginning of 2024 and early 2025, 86% were unvaccinated. However, preventing the transmission of the virus and of measles outbreaks necessitates a vaccination coverage of at least 95% with two doses of a measles-containing vaccine. Regrettably, the vaccination coverage in most European countries falls short of this critical target. Based on 2023 data, only four countries (Hungary, Malta, Portugal and Slovakia) have reported meeting this necessary threshold [6].

The persistent transmission of measles in Europe reflects the gaps in vaccination coverage and accumulation of susceptible individuals across different age groups in the population. For instance, a study from Austria featured in this edition of *Eurosurveillance* reports important immunity gaps among young and middle-aged individuals already before the COVID-19 pandemic in Austria [5]. Such gaps put certain population groups at risk, such as children who are either too young to receive vaccinations or people who cannot be immunised due to medical reasons and remain susceptible to measles. These vulnerable individuals rely heavily on widespread high vaccination coverage in the community to safeguard them from the disease.

Ensuring high vaccination coverage is key for preventing not only measles but also other vaccine-preventable diseases. A recent study published in the current issue was conducted in London following the detection of poliovirus in sewage in London in 2022 and showed the feasibility but also limitations of opportunistic poliovirus testing of residual stool samples from children seeking healthcare as rapid response in areas where poliovirus circulation is suspected [3]. This found a range of enteroviruses from groups A, B and C in the human samples, but no poliovirus. In Europe, between September and December 2024, five countries (Finland, Germany, Poland, Spain and the UK) reported detections of circulating vaccine-derived poliovirus type 2 (cVDPV2) in sewage samples, marking the first instances of such detections in European countries through environmental surveillance [10]. While most EU/EEA countries report national polio vaccination coverage above 90%, subnational data paint a more complex picture, with only 39% of reporting areas reaching the 90% threshold. This underscores the need for continuous evaluation of vaccination strategies at both national and sub-national levels. According to ECDC estimates for the EU/EEA, ca 600,000 children aged 12–23 months may not have completed a full primary polio vaccination course in 2022 and 2023 [11].

Reports of substantial increases in measles incidence or multiple detections of cVDPV, typically result in recommendations to close immunisation gaps in the population, verify vaccination status of individuals and ensure adherence to vaccination schedules. Despite these efforts, vaccination coverage for many vaccines continues to decline. One notable and worrisome signal is that there appears to be an increasing socioeconomic divide in vaccination timeliness and rates as pointed out in two articles in this issue. Following the birth cohorts 2015, 2017 and 2019 for 48 months to assess measles-mumps-rubella (MMR) vaccine dispensation timeliness after the introduction of vaccine mandates in France in 2018, Scronias et al. report that despite improvements in MMR vaccination, the vaccine was dispensed on average 7 months later than recommended for 33% of children in the 2019 cohort and for children from low-income families, they were dispensed even later (at least 1 month later compared with children from higher-income families). Moreover, independent of the COVID-19 pandemic, the French 2019 cohort still did not reach the 95% World Health Organization (WHO) target of two MMR doses, neither at 24 nor at 48 months of age. Another article by Jary et al. illustrates inequalities in vaccination in a measles outbreak disproportionally affecting deprived communities and some ethnic groups in Birmingham, England. It is encouraging that the MMR vaccination catch-up campaign was effective at targeting the most deprived populations. However, the authors state that “variations in MMR coverage gain occurred across ethnicities, with only small increases in MMR uptake in some ethnic groups that were most impacted by the outbreak” [2]. Socioeconomic inequalities in vaccination coverage have also been shown in reports from other countries in Europe and elsewhere [12,13].

To effectively address the challenge of low vaccination coverage, it is essential to consider the underlying contributing issues. While vaccine hesitancy is frequently highlighted as the primary reason, the articles from England and France show that reality is more complex. Several other factors play a role in suboptimal vaccination acceptance or uptake. They may include concerns over vaccine safety and effectiveness, a perceived lack of necessity due to low disease prevalence or severity, difficulties in accessing vaccines, socioeconomic disparities, and other related issues. When engaging with concerned individuals and population groups, social and behavioural science tools can help identify the key drivers of low vaccine uptake and enable targeted strategies to address these effectively.

Enhanced prevention of vaccine-preventable diseases amidst declining trust in science and scientific institutions, fuelled by the COVID-19 pandemic, necessitates a paradigm shift. As an EU public health organisation with the mission to improve the health of European citizens by protecting them from communicable diseases, the ECDC is taking this into consideration and has recently launched several initiatives aimed at improving vaccination uptake. The ECDC Prevention Framework integrates social and behavioural sciences, health promotion, health literacy and health education [14]. It also emphasises attention to socioeconomic risk factors to effectively address public health challenges. One such application of behavioural and social sciences to prevention is the 5C model [15], used to identify reasons for low vaccination acceptance and uptake in specific populations. This model provides a solid empirical foundation for interventions aimed at improving coverage. To fully realise the impact of social and behavioural sciences on preventing infectious diseases, it is essential to invest in expert capacity building. The ECDC Lighthouse community of practice, serves as an online platform for experts in communicable disease prevention to collaborate on integrating social and behavioural sciences into their public health efforts.

One key factor impacting vaccination rates are infodemics, a phenomenon characterised by an overload or lack of information, where some content may be accurate while a substantial portion is false or misleading. Infodemics can manifest both online and offline, during public health crises, or between them, eroding trust in clinical and public health services. Health information seekers are often faced with the need to navigate through the overabundance of online vaccination information, and the need to distinguish between facts and myths on countless websites and social media posts. In response to this challenge, the ECDC is actively addressing infodemics through an infodemics management programme.

Overall, to reverse the trend of decreasing vaccination coverage for key vaccines and of the increasing risk of vaccine-preventable disease outbreaks, a focus on health education and literacy of populations and investment in health promotion based on social and behavioural science insights is needed. Addressing the challenges of vaccination requires a concerted effort. In close collaboration with its partners, including other EU agencies such as the European Medicines Agency and the WHO, ECDC is committed to supporting countries in Europe in achieving optimal protection of their citizens against vaccine-preventable diseases. Only through collaboration and complementarity of approaches and applying principles of equity can we ensure optimal prevention and control of vaccine-preventable diseases in Europe.

## Note

Piotr Kramarz is Chief Scientist and Head of Unit Scientific Methods and Standards of ECDC and an Associate Editor of *Eurosurveillance*.

Ines Steffens is Editor-in-chief of *Eurosurveillance* and Head of the *Eurosurveillance* Editorial Office.

## References

[1] Heymer EJ Clark SA Campbell H Ribeiro S Walsh L Lucidarme J Use of 4CMenB vaccine in the control of an outbreak of serogroup B invasive meningococcal disease in an elderly care home, England, November 2023. Euro Surveill. 2025;30(16):2400673. 10.2807/1560-7917.ES.2025.30.16.2400673 PMC12023728 40276883

[2] Jary H Pullen A Howett D Hani E Suleman S Byrne L Sociodemographic inequalities in the epidemiology and vaccine uptake within a large outbreak of measles in Birmingham, England, 2023 to 2024. Euro Surveill. 2025;30(16):2400652. 10.2807/1560-7917.ES.2025.30.16.2400652 PMC12023730 40276885

[3] Rowland T Gopal R Patel M Celma C Campbell CNJ Machin N Community surveillance after detection of poliovirus in the environment in London, United Kingdom, October 2022 to April 2023. Euro Surveill. 2025;30(16):2500025. 10.2807/1560-7917.ES.2025.30.16.2500025 PMC12023727 40276887

[4] Scronias D Fressard L Fonteneau L Guagliardo V Verger P . Persistence of major socio-economic inequalities in childhood measles–mumps–rubella vaccination coverage and timeliness under vaccination mandates, France, 2015 to 2024. Euro Surveill. 2025;30(16):2400674. 10.2807/1560-7917.ES.2025.30.16.2400674 PMC12023726 40276886

[5] Springer DN Borsodi C Camp JV Redlberger-Fritz M Holzmann H Kundi M Seroprevalence against measles, Austria, stratified by birth years 1922 to 2024. Euro Surveill. 2025;30(16):2400684. 10.2807/1560-7917.ES.2025.30.16.2400684 PMC12023729 40276882

[6] European Centre for Disease Prevention and Control (ECDC). Measles-mumps-rubella monthly report. Stockholm: ECDC. [Accessed: 23 Apr 2025]. Available from: https://measles-rubella-monthly.ecdc.europa.eu/

[7] Texas Health and Human Services. Texas announces second death in measles outbreak. Austin: Texas Department of State Health Services; 6 Apr 2025. Available from: https://www.dshs.texas.gov/news-alerts/texas-announces-second-death-measles-outbreak

[8] Centers for Disease Control and Prevention (CDC). History of Measles. Atlanta: CDC; 9 May 2024. Available from: https://www.cdc.gov/measles/about/history.html

[9] Minta AA Ferrari M Antoni S Portnoy A Sbarra A Lambert B Progress toward measles elimination - worldwide, 2000-2022. MMWR Morb Mortal Wkly Rep. 2023;72(46):1262-8. 10.15585/mmwr.mm7246a3 37971951 PMC10684353

[10] Böttcher S Kreibich J Wilton T Saliba V Blomqvist S Al-Hello H Detection of circulating vaccine-derived poliovirus type 2 (cVDPV2) in wastewater samples: a wake-up call, Finland, Germany, Poland, Spain, the United Kingdom, 2024. Euro Surveill. 2025;30(3):2500037. 10.2807/1560-7917.ES.2025.30.3.2500037 39850005 PMC11914958

[11] European Centre for Disease Prevention and Control (ECDC). Assessing the risk to public health of multiple detections of circulating vaccine-derived poliovirus type 2 (cVDPV2) in wastewater in the EU/EEA. Stockholm: ECDC; 30 Jan 2025. Available from: https://www.ecdc.europa.eu/sites/default/files/documents/assessing-risk-public-health-detection-poliovirus-wastewater.pdf

[12] Mercogliano M Valdecantos RL Fevola G Sorrentino M Buonocore G Triassi M An ecological analysis of socio-economic determinants associated with paediatric vaccination coverage in the Campania Region: A population-based study, years 2003-2017. Vaccine X. 2024;18:100482. 10.1016/j.jvacx.2024.100482 38585381 PMC10997839

[13] Arat A Moore HC Goldfeld S Östberg V Sheppeard V Gidding HF . Childhood vaccination coverage in Australia: an equity perspective. BMC Public Health. 2021;21(1):1337. 10.1186/s12889-021-11345-z 34229652 PMC8261950

[14] European Centre for Disease Prevention and Control (ECDC). ECDC framework for prevention of communicable diseases and related special health issues. Stockholm: ECDC; 2024. Available from: https://www.ecdc.europa.eu/sites/default/files/documents/Prevention-framework-final.pdf

[15] Betsch C Schmid P Heinemeier D Korn L Holtmann C Böhm R . Beyond confidence: Development of a measure assessing the 5C psychological antecedents of vaccination. PLoS One. 2018;13(12):e0208601. 10.1371/journal.pone.0208601 30532274 PMC6285469

